# Non-invasive brain stimulation as therapy: systematic review and recommendations with a focus on the treatment of Tourette syndrome

**DOI:** 10.1007/s00221-021-06229-y

**Published:** 2021-10-13

**Authors:** Katherine Dyke, Georgina Jackson, Stephen Jackson

**Affiliations:** 1 School of Psychology, University of Nottingham, Nottingham, UK; 2 Institute of Mental Health, School of Medicine, University of Nottingham, Nottingham, UK; 3 School of Medicine, The University of Nottingham, Nottingham, UK

**Keywords:** Transcranial magnetic stimulation (TMS), Transcranial direct current stimulation (tDCS), Theta burst stimulation (TBS), Supplementary motor area (SMA), Tourette syndrome (TS), Tics

## Abstract

Tourette syndrome (TS) is a neurodevelopmental condition characterised by tics, which are stereotyped movements and/or vocalisations. Tics often cause difficulties in daily life and many with TS express a desire to reduce and/or gain control over them. No singular effective treatment exists for TS, and while pharmacological and behavioural interventions can be effective, the results are variable, and issues relating to access, availability and side effects can be barriers to treatment. Consequently, over the past decade, there has been increasing interest into the potential benefits of non-invasive brain stimulation (NIBS) approaches. This systematic review highlights work exploring NIBS as a potential treatment for TS. On balance, the results tentatively suggest that multiple sessions of stimulation applied over the supplementary motor area (SMA) may help to reduce tics. However, a number of methodological and theoretical issues limit the strength of this conclusion, with the most problematic being the lack of large-scale sham-controlled studies. In this review, methodological and theoretical issues are discussed, unanswered questions highlighted and suggestions for future work put forward.

## Introduction

In this systematic review, we explore the topic of non-invasive brain stimulation (NIBS) as a potential treatment, or adjunct to treatment, to help people with Tourette syndrome (TS) to reduce their tics. We consider the existing treatment options for TS, the justification and scientific reasoning behind using NIBS in this clinical group, and review the evidence to date. We also consider some limitations of frequently used therapeutic NIBS approaches and make suggestions for how this might usefully progress in the field of TS and beyond.

## Tourette syndrome

Tourette syndrome (TS) is a neurodevelopmental disorder that is found in the majority of cultures worldwide (Robertson et al. 2009). It affects approximately 1% of 5–18 years old (Cohen et al. 2013) and is approximately five times more common in males than females within this age group (Lichter and Finnegan 2015). TS is characterised by the occurrence of tics, which are repetitive, stereotyped movements and/or vocalisations of short duration, which can occur many times throughout a day. In some instances, motor tics can be physically painful and result in injury due to their strong and repetitive nature; tics can attract unwanted attention and be distressing. While tics, and the way that individuals cope with them are varied, they can influence many aspects of daily life including social, occupational/academic, and psychological well-being of both adults and children (Conelea et al. 2011, 2013). Consequently, many individuals with a diagnosis of TS will seek out strategies to minimise the occurrence of, or gain control over, their tics.

There are several factors which make developing effective treatments for TS challenging. First, the biological underpinnings of this condition are complex, multi-faceted, and not fully understood. TS rarely presents in isolation, and the majority of individuals with TS also meet the diagnostic criteria for at least one other neuropsychiatric condition, with the most prevalent co-morbidities being obsessive compulsive disorder (OCD) and attention deficit hyperactivity disorder (ADHD) (Wright et al. 2012). Subtly different changes in brain structure and function are likely to underlie these different symptom profiles, which in turn may influence response to therapeutic interventions. Sample sizes are often too small to thoroughly investigate this in humans; however, research in animals has compellingly linked regionally specific disruption in the striatum to tic-like behaviours, compulsions, and hyperactivity (see Bronfeld et al. (2013) for review).

Another challenge in developing treatments for TS is the practical and ethical considerations associated with the treatment of children/adolescents. For example, behavioural interventions often require a level of sustained attention and introspection, which can be difficult for younger children. There are also complex issues surrounding medication use in this age group, including consideration of potential side effects and the impact of sustained medication use on the developing brain. These issues are compounded by the fact that there is relatively little known about the developmental trajectory of this disorder, and it is unclear why some individual's tics reduce with age (Bloch and Leckman 2009), while for others, they do not.

## Treatment options for Tourette syndrome

Despite the numerous challenges, a number of tic reduction/management approaches are available. At present, the most common forms of treatment for tics are medication and behavioural therapy.

Habit reversal therapy (HRT) (Azrin and Nunn 1973) and extensions of this, such as comprehensive behavioural intervention for tics (CBIT) (Piacentini et al. 2010), are tic focused, and involve raising an individual’s awareness of sensory experiences prior to a tic and then encouraging the use of subtle, incompatible movements/vocalisations to be voluntarily executed until the need to tic reduces. Both HRT and CBIT are dependent on individuals being able to recognise subjective sensations often called premonitory urges (PU). PU are often described as feelings of discomfort or pressure prior to a tic, and are experienced by the vast majority of people with TS as an urge-to-tic (Bliss 1980). However, the experience of PU appears to vary with age and tic severity—in that older individuals and those with stronger tics have reported higher rates of PU (Sambrani et al. 2016). Interestingly, while both treatments have been found to be effective (Bate et al. 2011; Dutta and Cavanna 2013; McGuire et al. 2014), and appear to be dependent on the link between PU and tics, the evidence linking the two is inconclusive (Ganos et al. 2012) and much debated within the field (Cavanna et al. 2017; Jackson et al. 2011). Unfortunately, these types of therapy are not suitable or effective for all individuals, and access to specialists who are able to provide this treatment is often limited and remains an issue for many (Cuenca et al. 2015; Hollis et al. 2016). As a result, a common alternative to behavioural therapy is pharmacological treatment.

A systematic review found that over half of young people with TS had received medication to help with their tics (Hollis et al. 2016). While there is no single drug that directly targets tics, a range of medications can be effective in helping to reduce them. These include antipsychotics such as haloperidol which work by blocking D2 dopamine receptors, and alpha agonists such as clonidine which inhibit the release of noradrenaline (Kurlan 2014). Other drugs commonly prescribed to treat TS include atypical antipsychotics such as risperidone and aripiprazole (Hollis et al. 2016). Unfortunately, the side effects of these medications can outweigh the benefits for many individuals, as they include weight gain, stomach irritation, drowsiness, and sleep disturbances [see Hollis et al. (2016) for review].

For individuals who have disabling and treatment-resistant tics, deep brain stimulation (DBS) can be highly effective. Direct implantation of electrodes to regions of the basal ganglia, including the globus pallidus, the nearby internal capsule, or the centromedian nucleus of the thalamus, have been shown to substantially reduce tics, and can also reduce symptoms of co-morbid conditions such as OCD (Baldermann et al. 2016; Coulombe et al. 2019). However, this approach involves invasive brain surgery and its associated risks. There are also ethical considerations regarding the use of this type of intervention in children (Hedderly 2017; Servello et al. 2016), and again, there is very limited access to this form of treatment.

Other treatments include psychoeducation (Nussey et al. 2013), relaxation training (Bergin et al. 1998), and the use of botulinum toxin (Pandey et al. 2018), and have varying levels of effectiveness and various strengths and challenges when considering costs and benefits to individuals [for review, see Hollis et al. (2016)].

More recently, non-invasive brain stimulation (NIBS) has also been explored as a potential treatment for tics. NIBS possesses several possible advantages over other approaches. Given proper administration and monitoring, NIBS is largely free of adverse or side effects. NIBS can be used at rest without the need for cognitively demanding tasks (such as continuously monitoring PU) and can be used to non-invasively target-specific cortical regions associated with the pathophysiology of a condition. However, as will be discussed in the following sections, therapeutic use of NIBS has its own complexities, limitations, and areas for development.

## Non-invasive brain stimulation as a potential therapeutic intervention

The first studies exploring the use of NIBS as a potential therapeutic intervention for tics were conducted in the early 2000s. Since then, there has been a small but steady increase in studies exploring the therapeutic potential of forms of NIBS such as repetitive transcranial magnetic stimulation (rTMS) and transcranial direct current stimulation (tDCS).

### Brief introduction to rTMS and theta burst stimulation (TBS)

Transcranial magnetic stimulation (TMS) is a non-invasive brain stimulation technique that was first reported in 1985 (Barker et al. 1985). During stimulation, a brief but strong electrical current is delivered from an electrical capacitor to the TMS coil which contains conductive windings of wire. When an electrical current is present, a fluctuating magnetic field perpendicular to the coil is generated. The resultant magnetic fields can pass painlessly through the scalp and skull and induce an electrical current on the cortical surface. This induced current can then influence electrical signaling of neuronal populations. In particular, it can depolarize neurons or their axons (Hallett 2000). At present, the most commonly used TMS method for therapeutic neuro-modulation in patients with TS is rTMS, including a patterned version of this known as theta burst stimulation (TBS). Early rTMS studies typically involved exploring the effects of stimulation applied to the motor cortex by assessing changes in motor-evoked potentials (MEPs) (Chen et al. 1997; Pascual-Leone et al. 1994). The consensus from these and numerous subsequent studies is that low frequency rTMS (1 Hz) tends to lead to a reduction in cortical excitability (indexed by smaller MEPs), whereas higher frequencies (typically 5–20 Hz) are more often associated with faciliatory effects on corticospinal output (larger MEPs). While this is the general trend, it should be noted that substantial individual variability in response to these protocols is often observed, with differences in both the magnitude and direction of effects reported (Maeda et al. 2000).

TBS is a patterned form of rTMS, in which bursts of 3 TMS pulses at 50 or 30 Hz are delivered at a rate of 5 Hz. This patterned stimulation can be delivered either in a continuous (cTBS) or intermittent (iTBS) fashion (Huang et al. 2005). As with rTMS, it appears that both facilitation and inhibition of MEP amplitudes are possible following TBS stimulation, with cTBS reported to typically have a net inhibitory effect, while iTBS tends to result in net increased cortical excitability. However, as is the case for other forms of rTMS, both inter- and intra-individual variability has been reported in response to TBS (Lopez-Alonso et al. 2014; Ozdemir et al. 2021; Vallence et al. 2015).

Changes in cortical excitability, as indexed by alterations in MEP amplitude, suggest that the effects of a single session of rTMS and TBS can last over 30 min [for example: Jung et al. (2008); Wischnewski and Schutter (2015)]. These sustained aftereffects are frequently referred to as reflecting long-term potentiation (LTP) and long-term depression (LTD) like changes, which are likely caused by alterations in synaptic transmission. While this may be a slight oversimplification (see Lefaucheur et al. (2014) for discussion), it is nonetheless clear that the effects of such protocols can outlast the stimulation period, rendering them interesting as potential therapeutic interventions for TS.

### Brief introduction to tDCS

Another NIBS technique with potential as a treatment for tics is transcranial direct current stimulation (tDCS). TDCS involves running a low voltage current (typically between 1 and 2 mA) between a minimum of two electrodes, at least one of which is placed on the scalp. Like rTMS/TBS approaches, tDCS in healthy adults has been shown to induce changes in cortical excitability outlasting the stimulation period. The exact neurobiological mechanisms underpinning tDCS effects are not fully understood, but in general, it is accepted that effects during stimulation are likely related to alterations in membrane potential, whereas the aftereffects appear to be dependent on alteration to *N*-methyl-d-aspartate (NDMA) (Liebetanz et al. 2002; Nitsche et al. 2003) and α-amino-3-hydroxy-5-methyl-4-isoxazolepropionic acid (AMPA) (Martins et al. 2019; Stafford et al. 2018) receptor channels. Changes in levels of the inhibitory neurotransmitter gamma-aminobutyric acid (GABA) following tDCS have also been observed (Nitsche et al. 2004; Stagg et al. 2009). Typically, regions underneath the anode show a temporary increase in cortical excitability, reflected in increased MEP amplitude, whereas the opposite is true for areas under the cathode. As with rTMS and TBS, the patterns of response to stimulation vary, and substantial inter- and intra-subject variability in response to tDCS have been demonstrated (Dyke et al. 2016; Horvath et al. 2016; Wiethoff et al. 2014).

tDCS has some practical advantages over TMS. Specifically, it is relatively cheap to purchase, easy to administer, and is increasingly being explored as an intervention that is suitable for home administration for several conditions (Palm et al. 2018). However, this technique is less established, particularly in terms of therapeutic use in the treatment of TS.

### Identifying stimulation sites

One of the most important practical considerations in developing NIBS as an effective therapy is to identify appropriate sites for stimulation. While the exact underlying neurobiology of TS is not fully understood, dysfunction within cortical–striatal–thalamic–cortical (CSTC) circuits have been heavily implicated in the pathophysiology of tic disorders (Greene et al. 2015; Mink 2006). Many of the regions implicated (including the areas of the basal ganglia targeted by DBS) cannot be directly reached using NIBS, due to the restricted depth penetration of induced (rTMS/TBS) and direct weak electrical currents (tDCS). However, studies combining Magnetic Resonance Imaging (MRI) with TMS have revealed changes in blood-oxygenation-level-dependent (BOLD) in cortical and subcortical regions connected to the stimulation site (for review, see Fox et al. (2012)). For example, stimulation of the sensorimotor cortex using rTMS has been shown to induce BOLD changes in deeper structures such as the basal ganglia via inter-connected neural pathways (Bestmann et al. 2004, 2005). tDCS has also been shown to alter BOLD activity in regions which are functionally connected to the targeted cortical site of stimulation, (Saiote et al. 2013), with at least one study suggesting that modulation of deep structures such as the thalamus is possible (Polania et al. 2012). Consequently, NIBS methods for treating tics have centred on the idea of reducing cortical excitability within regions such as the supplementary motor cortex (SMA) and primary motor cortex (PMC), while also aiming to increase the engagement of inhibitory circuits and take advantage of connectivity between cortical regions and relevant deeper structures.

The SMA is a particularly appealing target site for therapeutic stimulation due to its role in modulating descending corticospinal projections, its likely involvement in tic production, and evidence of structural and functional alterations in TS. The SMA has extensive connections to areas relating to motor control and cognitive processing (Picard and Strick 2001), and work in primates has demonstrated SMA involvement in neural networks connecting cortical, thalamic, and basal ganglia pathways (Haber 2003); regions which are all heavily implicated in TS. The SMA of individuals with TS has been shown to exhibit altered metabolic activity (Eidelberg et al. 1997) and increased concentrations of the inhibitory neurotransmitter GABA (Draper et al. 2014). It has also been shown to be active immediately prior to tic onset (Bloch et al., 2016a; Bohlhalter et al. 2006; Hampson et al. 2009). The white matter pathways connecting the striatum and thalamus with PMC and SMA have been reported to be increased in TS, in a manner which correlates positively with symptom severity (Worbe et al. 2015). Furthermore, SMA connectivity to PMC during self-paced finger movements is altered in TS (Franzkowiak et al. 2012) and differences in patterns of activation in SMA/pre-SMA have been shown during Go/NoGo (Thomalla et al. 2014) and stop signal tasks (Ganos et al. 2014) relative to healthy controls.

Due to its prominent role in the motor system, direct targeting of the primary motor cortex (PMC) is another logical stimulation site for the therapeutic treatment of TS using NIBS approaches. The justification for this is largely based on work which suggests that at rest there is reduced inhibition within this area. This has typically been assessed by measuring short-interval intra-cortical inhibition (SICI). SICI is a paired pulse TMS technique in which a subthreshold conditioning pulse is applied to the motor cortex 1–5 ms prior to a supra (i.e., above) threshold test pulse. The first pulse modulates the effects of the second, typically causing a reduction in motor-evoked potentials when compared to those generated by the test pulse alone. SICI has been reported to be reduced in adults with TS (Heise et al. 2010; Orth et al. 2005, 2008; Orth and Rothwell 2009; Ziemann et al. 1997) and to correlate with motor tics and ADHD symptoms in both children and adults (Gilbert et al. 2004, 2005). Animal models and pharmacological studies have suggested that the effects of SICI are strongly mediated by GABAergic activity, with emphasis on the involvement of GABA-A receptors (Hanajima and Ugawa 2008; Ziemann 2013), and a minor contribution from GABA-B receptors (McDonnell et al. 2006). Hence, these findings suggest that GABAergic synaptic inhibition within primary motor regions is reduced in TS.

Theoretically, it may be possible to alter the balance of excitation/inhibition within cortical regions such as the SMA and PMC using repeated application of NIBS. Specifically, as the majority of previous research suggests that these regions are overly responsive in TS, researchers have used approaches which when assessed in healthy adults have been reported (on average) to reduce cortical excitability (cathodal tDCS, _c_TBS, and 1 Hz rTMS).

## Systematic review of the literature

### Search strategy

**Fig. 1:**
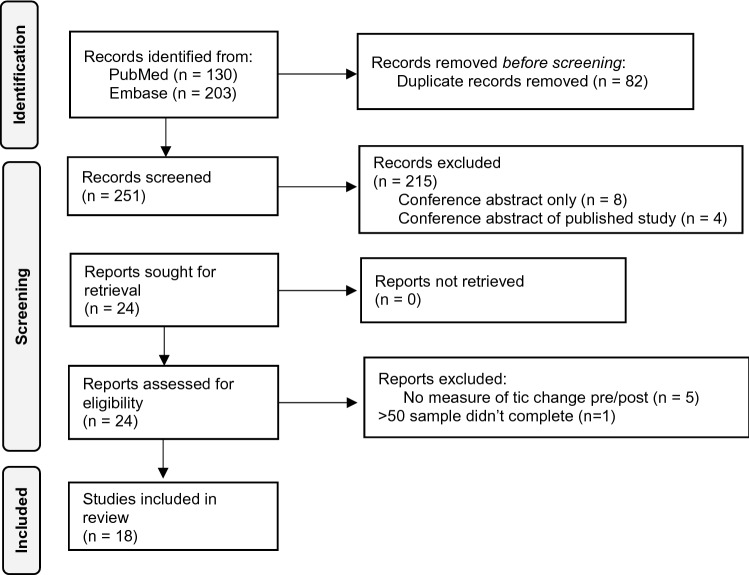
Schematic of systematic review using PRISMA approach

The approach to systematic review was informed by the guidelines outlined in the preferred reporting items for systematic review and meta-analysis (PRISMA) (Page et al. 2021) (Fig. 1).

We searched PubMed and Embase using the following key terms and Booleans: (‘tic’ OR ‘Tourette*’ OR ‘tic disorder’) AND (‘transcranial magnetic stimulation’ OR ‘theta burst stimulation’ OR ‘transcranial direct current stimulation’ OR ‘transcranial alternating current stimulation’ OR ‘transcranial random noise stimulation’ OR ‘TMS’ OR ‘tDCS’). This search was carried out without search restrictions from the first date available until 30th June, 2021 (including articles only available online at the time of the search).

### Eligibility criteria

The studies included in the review had to meet the following criteria: (a) participants must have Tourette’s, tics, or tic disorder; (b) a form of non-invasive brain stimulation must have been used; (c) outcome measures must include a measure of change in tics (e.g., YGTSS or video recording) before and at least once after stimulation; (d) an English version of the article must be available.

The following types of entry were excluded: (a) reviews; (b) animals studies; (c) conference abstracts/presentations; (d) studies in which > 50% of participants did not complete trial. Letters to editor and case studies were included, providing that they met the other criteria.

### Information extraction

The following information was extracted from studies meeting the eligibility criteria: study type (open label, randomised control, and cross over); participant demographics (number, age, sex, and diagnosis); NIBS method (stimulation type, are of stimulation, pulse type/intensity); number of sessions tested; measurement(s) used to assess symptom change; key findings and statistics. Information extraction and assessment of articles for inclusion was completed independently by KD without use of automation tools.

## Results of systematic review

In total, our search criteria yielded 333 articles. Duplicates from the two sources were manually identified and removed (*n* = 82) leaving a sample of 251 records to be screened. The lead author (KD) screened the titles and abstracts of the remaining articles using the eligibility criteria, which resulted in 24 articles for retrieval. Of these, a further 6 were excluded; 5 as they did not involve pre/post measures of tic change (Marsili et al. 2017; Sun et al. 2020; Suppa et al. 2011; Suppa et al. 2014; Wu and Gilbert 2012) and 1 additional case study as the participant withdrew early (Salatino et al. 2014).

The authors were aware of a further two articles (Carvalho et al. 2015; Tajadini et al. 2018) which were not identified in the systematic review, but are nevertheless relevant to this topic. Inclusion of these two articles takes the total sample size up to 20, of which 13 studies used rTMS/TBS (Table 1) and 6 used tDCS. It should be noted that two publications (Munchau et al. 2002; Snijders et al. 2005) relate to the same study and hence are summarised together in Table 1.

## Evidence so far: rTMS and TBS as a treatment for tics

The measures used to assess changes in tics following NIBS vary from study to study, with one general exception—the Yale Global Tic Severity score [YGTSS (Leckman et al. 1989)]. The YGTSS is a semi-structured interview used to rate the number, frequency, intensity, complexity, and interference of the motor and phonic tics experienced by an individual in the past week. The scale is commonly used within clinical assessment of, and research into TS, and has been shown to have good psychometric properties (Leckman et al. 1989; Storch et al. 2005). The following sections primarily focus on change in tics as assessed by YGTSS scores, specifically total tic severity score which is calculated from the sum of the subscales. A more extensive summary of the effects of NIBS on other measures including assessment of common co-morbidities using the Yale-Brown Obsessive Compulsive scale [Y-BOCS (Scahill et al. 1997)] and Attention Deficit Hyperactivity Disorder Rating Scale [ADHD_RS (DuPaul et al. 1998)] can be found in Tables 1 and 2.

**Table 1:** Studies exploring TMS as a potential therapeutic intervention for TS

Study	Study type	Sample size	Participant age (mean ± SD)/sex	Diagnosis	TMS: location, type, intensity, pulses per session	Sessions	Measurement	Summary of main results
Kwon et al. (2011)	OLT	10	Age: 9.57 ± 2.75 M/F: 10/0	TS (4), TS + ADHD (3), TS + MDD (2), TS + OCD (1)	Bilateral SMA 1 HZ rTMS, 100% RMT, 1200	10	YGTSS; CGI; CDRI	Significant reduction in scores for YGTSS (*F* = 0.788, *p* = 0.012) and CGI (*F* = 18.09, *p* = 0.002)
Le et al. (2013)	OLT	25	Age: 10.61 ± 2.18 M/F: 22/3	TS(25), no further details	Bilateral SMA 1 HZ rTMS, 110% RMT, 1200	20	YGTSS; CGI;SNAP_IV;CDI; SCAS; RMT	Significant reduction in scores for YGTSS (*F* = 44.667, *p* < 0.001); CGI (*F* = 42.875, *p* < 0.001); SNAP_IV (*F* = 6.828, *p* = 0.005); CDI (*F* = 7.44, *p* = 0.001) and SCAS (*F* = 8.036, *p* = 0.003) Significant increases in Left RMT (*F* = 29.263, *p* < 0.001) and right RMT (*F* = 22.466, *p* < 0.001)
Mantovani et al (2007)	OLT	2	Age: 22 and 16 M/F:2/0	TS + OCD + ADHD + MDD (2)	Bilateral SMA 1HZ rTMS, 110% RMT, 1200	10	YGTSS; Y-BOCS; CGI; ADHD-RS; HDRS; HARS	Reduction in scores for YGTSS (Participant 1: 36%; Participant 2: 68%); Y-BOCS (P1: 28%, P2: 16%); CGI (P1: 42%, P2: 50%); ADHD-RS (P1: 7%, P2: 72%); HDRS: (P1: 71%, P2: 67%) & HARS: (P1: 63%; P2: 100%)
Mantovani et al. (2006)	OLT	10	Age: 33.5 ± 13.5 M/F: 8/2	TS (3), OCD (5), TS + OCD(2)	Bilateral SMA 1 Hz rTMS, 100% RMT, 1200	10	YGTSS; CGI; YBOCS; HDRS; HARS; SCL; BDI; SAD; SASS	Significant reduction in YGTSS scores for participants with TS/TS + OCD (*F* = 10.707, *p* = 0.005) Subgroup analysis by group (TS, TS + OCD, OCD) show varying levels of reduction in scores for YBOCS, CGI, HARS, HDRS, SAD, BDI, sSCL-90 and SASS
Bloch et al. (2016a)	OLT	12	Age: 32.6 ± 12.7 M/F: 6/6	TS (2), TS + OCD (6) TS + ADHD (4)	Bilateral SMA 1 Hz rTMS, 110% RMT, 1200	20	YGTSS, YBOCS, CGI, SASS, HDRS, HARS, QIDSSR	No significant change in YGTSS (*F* = 1.26, *P* = 0.302); YBOCS (*F* = 3.95, *p* = 0.302) and CGI (*F* = 8.53, *p* = 0.005) Significant decrease in HDRS (*F* = 4.47, *p* = 0.029) Subgroup analysis for TS + OCD sample (*N* = 6) show significant reduction in YGTSS (*F* = 4.65, *p* = 0.037) and YBOCS (*F* = 5.47, *p* = 0.035)
Landeros-Weisenberger et al. (2015)	RCT, double, parallel and OLT	Active 9 Sham 11	Active Age: 29.1 ± 7.4 M/F: 7/2 Sham Age: 37.5 ± 12.9 M/F: 2/9	Active TS (1), TS + OCD (3), TS + ADHD(5) Sham TS (6), TS + OCD (2), TS + ADHD (3)	Bilateral SMA/sham 1 Hz rTMS, 110% RMT, 1800	15 (RCT) + 15 (OLT)	YGTSS; CGI; PUTS; YBOCS; ASRS	RCT phase: NS differences between YGTSS scores for sham (31.5 ± 8.1) and active (29.5 ± 11.9) groups. NS change for CGI, PUTS, YBOCS or ASRS Subgroup analysis for active rTMS in RCT + active rTMS in OLT (*N* = 7) showed significant reduction in YGTSS (*t* = 2.6, *p* = 0.04)
Wu et al. (2014)	RCT, double, parallel	Active 6 Sham 6	Active Age: 13.5 ± 3.9 M/F: 6/0 Sham Age: 15.5 ± 4 M/F: 3/3	Active TS(1), TS + ADHD(1), TS + OCD + ADHD (4) Sham TS (2), TS + OCD (1), TS + OCD + ADHD(3)	Bilateral SMA/sham cTBS, 3 pulse 30HZ bursts, repeated 5 times per second. 90% RMT. 600 pulses per train. 4 trains per day	2	YGTSS; PUTS; CYBOCS; finger tapping fMRI task; video	YGTSS reduction for sham (26.8 ± 4.8–21.7 ± 7.7) and active (27.5 ± 4.8–23.2 ± 9.8); but NS YGTSS group differences (*F* = 0.068, *p* = 0.43) NS group differences for video-based tic assessment (*F* = 2.01, *p* = 0.16) or other clinical measures (PUTS, CYBOCS) Significantly less activity during fMRI finger tap following cTBS within SMA (*p* = 0.02), left M1 (*P* = 0.004) and right M1 (*p* < 0.001)
Singh et al. (2018)	OLT	3	Age: 24, 18,15 M/F: 2/1	TS (1), TS + OCD (2)	Bilateral SMA 1 Hz rTMS, 110% RMT, 900	20	YGTSS,Y-BOCS	Varying reductions in YGTSS scores of 10, 50, and 65%. Y-BOCS reductions of 50% in one TS + OCD participant but increase of 13% for another
Kahl et al. (2021)	OLT	10	Age: 11:4 ± 1.8 M/F: 8/2	TS + ADHD (5), TS + ADHD + OCD (1) TS_ADHD + OCD + anxiety (1) TS + OCD_epilepsy + anxiety (1) TS + anxiety(1) TS + ADHD + anxiety + ODD	Bilateral SMA (each hemisphere separately) 1 Hz rTMS 100%RMT 900 pulses per hemisphere	15	YGTSS; neuro metabolite change (measures by MRS and TMS approaches); TMS motor maps; MASC 2; CDRS_R	Significant reduction in YGTSS score (*p* < .006, Cohens *d* = 2.9), MASC 2 (*p* < .005) and CDRS_R (*p* < 0.005) NS change in MRS measured neuro metabolites For TMS measures: Cortical silent period in non-dominant hemisphere increased (*t* = − 2.7, *p* = 0.035). Inter hemispheric facilitation in non-dominant hemisphere also increased (*t* = − 2.7, *p* = 0.028) NS change in motor maps or other TMS measures
Orth et al. (2005)	RCT, Single, Cross over	5	Age: 19–52 M/F: 4/1	TS (5)	A: L pre-motor/R pre- motor B: L pre-motor/R sham C: L sham/R sham 1 Hz rTMS, 80%AMT, 1800	2 (A) 2 (B) 2 (C)	YGTSS; MOVES; video	NS change (*p* > 0.05) for YGTSS; MOVES and video analysis for any condition (A, B or C)
Munchau et al. (2002) See also: Snijders et al. (2005) for further analysis using video data	RCT, Single, cross over	16	Age: 38 ± 13.2 M/F: 12/4	TS (9), TS + OCD (7)	A: L motor B: L pre-motor C: Sham 1 Hz rTMS, 80%AMT, 1200	2 (A) 2(B) 2(C)	YGTSS; MOVES; Hospital anxiety and depression scales; video	NS change (*p* > 0.05) for YGTSS; MOVES; anxiety or depression scales Video data show reduction in tics following each stimulation, but no significant differences between stimulation site (*p* > 0.05)
Chae et al (2004)	RCT, single, cross over	8	Age: 34.9 ± 16.4 M/F: 5/8	TS(2), TS + ADHD(1), TS + OCD(1), TS + OCD + ADHD(1), TS + LD(1), TS + OCD + MDD(2)	A1: L pre-frontal cortex, 1 Hz A2: L pre-frontal cortex 15 Hz B1: L Motor cortex, 1 Hz B2: L Motor cortex, 15 Hz C: Sham 120% rMT, 2400	1 (A1) 1 (A2) 1 (B1) 1 (B2) 1 (C)	YGTSS; CGI; Y-BOCS; video	NS trend towards YGTSS reduction (*t* = 2.2, *p* = 0.066) NS change in tics assessed by video (*z* = 0.67, *p* = 0.5) CGI significantly reduced (*z* = 2.0, *p* = 0.041) YBOCS decreased (*t* = 3.5, *p* = 0.01) NS effects of stimulation type for any measure (*p* > 0.05)
Fu et al (2021)	RCT, double, parallel	Active 15 Sham 15	Active Age: 19.7 ± 3.4 M/F: 3/12 Sham Age: 19.8 ± 4.4 M/F: 3/12	Active TS (15) Sham TS (15)	Active Bilateral parietal lobe (P3/ P4) 0.5 Hz, 400 pulses in three trains (1200 pulses per hemisphere) 90% rMT Sham Identical but 10% rMT	10	YGTSS; PUTS; video	Significant reduction in YGTSS scores (*f* = 192.22, *p* = < 0.001) Significant difference between groups (*f* = 26.268, *p* = < 0.001) Significant reduction in PUTS scores (*f* = 97.6, *p* = < 0.001) Significant difference between groups (*f* = 8.136, *p* = 0.008) Significant reduction in video analysis of tics (*f* = 220.8, *p* = < 0.001) Significant difference between groups (*f* = 19.18, *p* = < 0.001)

*ASRS* Adult Self-Report Scale, *ADHD_RS* Attention Deficit Hyperactivity Disorder Rating Scale, *BDI* Beck Depression Inventory, *CDRS-R* Children's Depression Rating Scale-Revised, *CDI* Children’s Depression Inventory, *CGI* Clinical Global Impression, *HDRS* Hamilton Anxiety Rating Scale, *HDRS* Hamilton Depression Rating Scale, *CDI* Kovacs Children’s Depression Inventory, *LD* learning disorder, *MRS* magnetic resonance spectroscopy, *MDD* major depressive disorder, *MOVES* Motor and Vocal tic Evaluation Survey, *MASC 2* Multidimensional Anxiety Scale for Children, Second Edition, *ODD* oppositional defiant disorder, *OLT* open-label trial, *QIDSSR* quick inventory of depressive symptomology self-report, *RCT* randomised-controlled trial, *SAD* scale for auto-evaluation of depression, *SASS* social adaptation self-evaluation scale, *SNAP-IV* Swanson, Nolan and Pelham Rating Scale, *SCL* symptoms check list, *SCAS* Spenser Children’s Anxiety Scale, *YBOCS* Yale-Brown Obsessive–Compulsive Scale, *YGTSS* Yale Global Tic Severity Scale

**Table 2:** Studies exploring tDCS as a therapeutic intervention for TS

Study	Study type	Sample size	Participant age (mean ± SD)/sex	Diagnosis	tDCS: electrode location, intensity, duration	Sessions	Measurement	Summary of main results
Carvalho et al. (2015)	OLT	1	Age: 16 M/F: 1/0	TS (1)	Cathode: pre-SMA Anode: upper deltoid 1.4 mA, 30 min	10	YGTSS; video; fMRI resting state	Reduction in YGTSS of 41% following stimulation, sustained at 6 month follow-up Decreased activity in left precentral and left cerebellum regions of sensorimotor resting-state network
Mrakic-Sposta et al. (2008)	RCT, single, cross over	2	Age: 26 and 31 M/F: 2/0	TS (1)	Cathode: left M1 Anode: upper deltoid A: 2 mA, 15 min B: sham, 15 min	5 (A) 5 (B)	YGTSS; Video; VAS	Reduction in YGTSS of 20% and 10% following active stimulation Reductions in YGTSS of 5% and 0% following sham stimulation No reduction in video assessed tics following sham, but approx. 60% reduction in motor tics following cathodal
Behler et al. (2018)	OLT	3	Age: 55, 20 and 18 M/F: 2/1	TS(1), TS + OCD(2)	Cathode *2: pre-SMA/SMA Anode: sternocleidomastoid muscle 2 mA, 30 min, Twice a day	10	YGTSS; video; Y-BOCS; PANAS	Reduction in YGTSS for one participant (34.5%) increase for other two (13% and 5%) Reduction in Y-BOCS for one participant (83%), no change for one and 20% increase for another
Eapen et al. (2017)	OLT (pilot)	2	Age: unknown M/F: unknown	TS (2)	Cathode: SMA Anode: right deltoid A: 1.4 mA, 20 min B: sham	18 (A) 9 B (B)	PUTS; ATQ	Reductions in ATQ by 35% and 21% following 6 weeks active stimulation Reductions in PUTS by 8% and 21% following 6 weeks active stimulation
Dyke et al. (2019)	RCT, single, cross over	10	Age: 22.8 ± 5.6 M/F: 5/5	TS (3), CTD (1), TS + ADHD (3), TS + anxiety (1), TS + OCD + depression (1)	Cathode: SMA Anode: right side of forehead A: 1 mA, 20 min B: Sham	1 (A) 1 (B)	TMS—recruitment curve; MEP amplitude; video data	No change in TMS measures (*p* > 0.05) Significant difference post sham vs post active (*t* = 2.35, *p* = 0.04) NS interaction between time (pre/post) and condition (active/sham) (*f* = 0.009, *p* = 0.93)
Tajadini et al. (2018)	OLT	1	Age: 31 M/F: 1/0	TS (1)	Cathode *2: left motor and left inferior frontal regions Anode: right side of forehead 2MA, 30 min, twice daily	10	VAS	Reductions in tics which were sustained for up to a year following stimulation

*ASRS* Adult Self-Report Scale, *ADHD_RS* Attention Deficit Hyperactivity Disorder Rating Scale, *BDI* Beck Depression Inventory, *CGI* Clinical Global Impression, *HDRS* Hamilton Anxiety Rating Scale, *HDRS* Hamilton Depression Rating Scale, *CDI* Kovacs Children’s Depression Inventory, *OLT* Open label trial, *PNAS* positive and negative affect Schedule, *QIDSSR* quick inventory of depressive symptomology self-report, *RCT* randomised-controlled trial, *SNAP-IV* Swanson, Nolan and Pelham Rating Scale, *SCL* symptoms check list, *SAD* scale for auto-evaluation of depression, *VAS* Visual analogue scale, *YBOCS* Yale-Brown Obsessive–Compulsive Scale, *YGTSS* Yale Global Tic Severity Scale

Several studies have reported significant reductions in YGTSS scores (indicating reduced tic severity) following rTMS/TBS stimulation applied bilaterally to the SMA using pulse configurations associated with reducing cortical excitability (e.g., 1HZ rTMS at 100–110% RMT). One such study conducted by Kwon et al. (2011) reported significantly reduced YGTSS scores in 10 children with TS, following 10 sessions of SMA stimulation. Tic reductions were still apparent at a 12 week follow-up and measures of general well-being were also significantly improved. A similar finding was published by Le et al. (2013) who reported a significant reduction in YGTSS scores following 20 days of rTMS in 25 children (that lasted up to 6 months in some cases); and by Mantovani et al. (2006) who reported significant reductions in YGTSS scores following 10 days of stimulation in 5 adults/adolescents, in addition to reductions in measures of OCD symptoms (Y-BOCS). In a smaller scale study with two participants, Mantovani et al. (2007) found reductions in YGTSS, Y-BOCS, and measures of ADHD (ADHD-RS) following 10 sessions of 1 Hz rTMS at 110%RMT over the SMA. Using similar parameters Singh et al. (2018) also reported substantial decreases in YGTSS scores in two third of participants. An additional study by Blochet al. (2016b) failed to find significant changes in YGTSS measures in 12 participants following 20 sessions of 1 Hz rTMS; however, in a subgroup of 6 participants who also had OCD, significant reductions in both YGTSS and YBOCS were found. More recently, a study by Kahl et al. (2021) demonstrated significant reductions in YGTSS scores in 10 children following 15 sessions of SMA stimulation.

Although the research discussed above appears promising, it should be noted that a few sham-controlled studies have yet been conducted. One notable exception is a study by Landeros-Weisenberger et al. (2015), in which the effects of 15 days of 1HZ rTMS on YGTSS scores were shown to be similar between sham stimulation and active stimulation conditions. Interestingly, the subset of participants who experienced an additional 3 weeks of active stimulation as part of the open-label phase of the study went on to show significant changes from baseline. At the time of writing, a single additional sham-controlled study targeting the SMA had been published by Wu et al. (2014), in which six participants experienced continuous theta burst stimulation (cTBS) over 2 days and six experienced sham stimulation. No significant differences in tic severity measured by YGTSS were found. Interestingly, fMRI BOLD response during a simple finger tapping task was significantly reduced over the SMA and bilateral primary motor cortex after cTBS, suggesting that rTMS had an inhibitory effect on the targeted SMA that spread to the primary motor cortex, but this did not affect tics.

rTMS studies targeting motor, pre-frontal, and pre-motor regions have yielded far less promising results. In three sham-controlled cross-over studies, rTMS applied to these regions was reported to produce no significant change in tic severity when compared to a sham control; (Chae et al. 2004; Munchau et al. 2002; Orth et al. 2005; Snijders et al. 2005). Although this may suggest that these targets are less effective, it is important to note that these studies used substantially fewer sessions than those often used in SMA studies, and that there are several methodological differences between studies (please refer to Table 1 for details).

Recently, a single study has been published in which the parietal lobe was targeted bilaterally over ten sessions (Fu et al. 2021). This study reports impressive reductions in YGTSS scores and video analysis of tics; including significant differences between sham and active groups. Neuroimaging work with fMRI has implicated the parietal lobe’s potential involvement in tic production (Bohlhalter et al. 2006; Neuner et al. 2014) and it may be that this site is a viable and promising stimulation site.

## Evidence so far: tDCS as a treatment for tics

Research exploring the use of tDCS therapeutically in individuals with TS has so far focused on protocols in which the cathode is placed over the SMA or motor cortex with the aim of reducing cortical excitability (see Table 2 for details). This has shown some therapeutic promise. For example, in a single-case study, Carvalho et al. (2015) found that tic severity (YGTSS) was reduced by 46% following ten sessions of cathodal tDCS over the pre-SMA. These effects were still present at a 6-month follow-up, and changes in resting-state networks were also identified. A similar result was demonstrated in a case study by Mrakic-Sposta et al. (2008) who also reported a reduction in tics following 5 days of cathodal tDCS applied to the left pre-motor cortex in two participants relative to a sham control condition. In addition, Eapen et al. (2017) reported reductions in tic and urge symptoms in two participants who experienced cathodal SMA stimulation for 6 weeks; and a single-case study by Tajadini et al. (2018) demonstrated sustained, beneficial, effects of cathodal stimulation over motor/inferior frontal regions. However, others have found only weak support for the beneficial effects of this approach. A recent study with three participants reported only 1 of 3 showed reduced tic severity after 10 sessions of cathodal pre-SMA stimulation (Behler et al. 2018). In addition, recent work has shown a small difference between tic frequency/intensity following SMA cathodal stimulation compared to sham stimulation, but no interaction and no clear effects of changes in cortical excitability (Dyke et al. 2019).

Overall, the evidence for the effectiveness of non-invasive brain stimulation to treat tics is rather mixed. Studies have mainly targeted cortical areas that are part of the cortico-striatal-thalamo-cortical loop and have attempted to attenuate tics by reducing the net excitability of these areas. However, a few studies have measured NIBS-induced changes within those targeted brain regions, and hence, it is unclear if the desired down-regulation of targeted areas was actually achieved and to what extent any relationships exist between physiological change in these regions and alteration in tic severity. This and several other important factors need to be considered when assessing the literature presented in Tables 1 and 2 and considering the future of NIBS as a therapeutic intervention for TS.

## Considerations for the future work

Here, we consider some of the caveats to previous NIBS research and make suggestions for future developments in the field. We focus on the use of NIBS for the treatment of tic disorder and the results presented in Tables 1 and 2. However, we note that many of these considerations are applicable more widely to the general study of NIBS as a therapeutic intervention for neuropsychiatric conditions.

### Study design

Of the 13 TBS/rTMS studies identified by this review, 7 are exclusively open label, and most of which report significant reductions in YGTSS scores following stimulation (see Table 1). Disappointingly, of the five studies targeting motor regions (SMA/M1) and including a sham control, none have provided convincing evidence for a beneficial effect of active stimulation. The study by Wu et al. (2014) is an interesting exception. Although no significant differences between sham and active conditions were found when assessing change in YGTSS scores, active stimulation resulted in significantly lower activation of SMA, left, and right M1 during a simple finger tapping task in comparison to sham. This is one of the few studies to attempt to record physiological change following NIBS, and although it is unclear why this did not translate into a larger reduction in YGTSS for the active group, it is an example of the type of study design which may be needed to further understand NIBS therapeutic potential.

At the time of writing, the most recent therapeutic trial of rTMS conducted by Fu et al. (2021) reported significant difference between YGTSS scores and video analysis of tics following bilateral stimulation of the parietal lobe. Unlike the previous sham-controlled trials discussed above, the difference between sham and active groups was found to be statistically different. This warrants further exploration and replication, but suggests that exploring different stimulation sites could be beneficial.

Although the majority of the results of sham-controlled studies using rTMS/TBS are discouraging, it is important to consider the potential impact of small sample sizes and methodological differences, including the age range of the participants (which tended to be older), stimulation site, and outcome measure (see separate section). It is clear that there is a need for large-scale randomised-controlled studies to be conducted. This recommendation is also true for tDCS studies. Of the studies reviewed, two employed a sham control (Dyke et al. 2019; Mrakic-Sposta et al. 2008). Encouragingly, both reported larger reductions in video recorded tics following cathode stimulation in comparison to sham. However, as previously noted, in Dyke et al. (2019), no significant interaction between tDCS condition and time of measure (pre/post) was found.

### NIBS in the brain at rest or during task relevant engagement?

The work reviewed in Tables 1 and 2 consists of studies conducted exclusively, while participants were at rest. In these studies, participants were typically asked to try to relax during stimulation and given no further task. The approach to applying NIBS at rest is worthy of consideration for three reasons. First, as with any TS study, the degree to which a true relaxed or rested stated is achieved is difficult to conclude, as there is always the possibility that participants are engaged in varying levels of tic suppression. Second, there is increasing evidence that the physiological effects of NIBS are influenced by cortical state at the time of stimulation (Hsu et al. 2016; Silvanto and Pascual-Leone 2008). Thirdly, depending on the mechanism through which NIBS approaches work, there might be substantial benefits to stimulation being delivered during or shortly after a participant engages in a task that activates symptom-relevant neural circuits.

Relating to the first and second point made above, tic suppression has been shown to cause transient alterations in motor cortical excitability as measured by TMS (Ganos et al. 2018) and several lines of converging evidence suggest that tic suppression influences multiple neural circuits (Ueda et al. 2021). Yet, controlling for this is challenging, and is often not discussed in NIBS-focused papers. This is noteworthy, as baseline cortical states have been shown to interact with subsequent NIBS-induced changes in cortical excitability. This is most convincingly demonstrated in motor regions, particularly by meta-plasticity priming studies. For example, the effects of a single session of rTMS appear to reverse when two periods of identical stimulation are applied in close temporal proximity (Fricke et al. 2011; Monte-Silva et al. 2010; Siebner et al. 2004), and priming motor regions using voluntary hand movements has also been shown to interact with the expected effects of stimulation [see (Karabanov et al. 2015)]. These studies highlight the complex interaction between baseline cortical activity states and NIBS, which are particularly pertinent when used to study TS ‘at rest’, given that this could mean vastly different things for different participants. Researchers should be mindful of the potential effects of factors such as tic suppression, and give participants clear and consistent instructions relating to this.

All the studies reviewed here use NIBS approaches which (on average) reduce cortical excitability in healthy adults when applied at rest. This approach is largely underpinned by the assumption that a reduction in cortical excitability during/shortly after stimulation is necessary for clinical effects; however, this is rarely measured or reported. In a recent study, we found that despite small improvement in tics immediately after cathodal tDCS, there was no evidence of reduced cortical excitability or alterations in GABA-A synaptic inhibition (Dyke et al. 2019). Alterations in cortical excitability following cathodal tDCS are also not consistently found in healthy adults (Dyke et al. 2016; Wiethoff et al. 2014). Yet, the relationship between the direction/magnitude of change in cortical excitability following NIBS on target outcomes (e.g., task modulation in healthy adults/clinical outcomes in TS) has not been systematically explored. Without this information, it is tempting to conclude that applying approaches which result in the largest reduction in cortical excitability will lead to the best clinical outcomes. However, depending on the exact underlying mechanisms through which NIBS approaches work, this is not necessarily the case.

Pharmacological studies exploring the effects of rTMS have implicated NDMA receptors which are known to be linked to LTP/LTD-type effects (Huang et al. 2007), and evidence for the involvement of these receptors has also been reported for tDCS aftereffects (Liebetanz et al. 2002; Nitsche et al. 2003). It follows from this that therapeutically useful changes may occur due to the stimulated region having strengthened/weakened synaptic connections. However, another interpretation of what happens during NIBS is that stimulation adds neural ‘noise’ and makes synaptic connections more variable, and hence more likely to change in response to inputs, rather than simply making them stronger (LTP like) or weaker (LTD like). A recent study by Kozyrev et al. (2018) found that high-frequency (10 Hz) rTMS in cats increased cortical excitability and variability which, when paired with training using visual stimuli, enabled reorganisation of visual maps. This effect was not observed in a sham control condition. Following stimulation, these normally well-defined visual maps became more varied, in that sensitivity to the orientations of visual stimuli were decreased. This finding is particularly striking as it suggests that NIBS is creating a period of enhanced plasticity, during which modulation of well-established neural responses is possible, and can be sustained for several hours. If the effect of NIBS is more akin to the addition of noise to sensorimotor circuits, then the average direction of change in cortical excitability may actually be of little importance. Further work is needed to understand how direction and magnitude of change in cortical excitability relate to alterations in learning/plasticity. However, the findings reported by Kozyrev et al. (2018) suggest a potential benefit to combining NIBS with a therapeutically relevant task. Future work exploring therapeutic use of NIBS in TS may consider using NIBS as an adjunct to therapy, for example, pairing stimulation with a task such as tic suppression or habit reversal, and monitoring changes in cortical excitability to better understand the underlying effects of NIBS and how these can be optimised.

### Participant demographics, intra-subject variability, and identifying biomarkers

Studies exploring the therapeutic potential of NIBS in TS have tended to be conducted using very small sample sizes (see Tables 1 and 2). Furthermore, effect sizes are rarely reported and individual data are not routinely shown. Small sample sizes are a common problem more generally and have been raised previously as a contentious issue within the field (Heroux et al. 2017; Minarik et al. 2016; Polania et al. 2018). This could be particularly problematic for the research discussed here, given the heterogeneous nature of the participants, who vary in age, medication use, and co-morbidities, amongst other factors. This is particularly noteworthy given that even within relatively homogeneous groups, variability in response to NIBS is often reported.

Variability in response to NIBS likely depends on several factors including genetics, age, cortical excitability prior to stimulation, and medication use (Ridding and Ziemann 2010). While samples are typically too small for sufficient subgroup analyses, a recent review paper summarising 11 studies of rTMS/TBS found that the benefits of stimulation were the largest in younger individuals with concurrent ADHD (Grados et al. 2018), a meta-analysis conducted at a similar time also indicated larger outcomes in studies with younger participants (Hsu et al. 2018). However, the reliability of these findings and reasons for this are unclear and warrant additional research.

A further important consideration is whether knowledge gained from studying the effects of NIBS in healthy participants is necessarily applicable to the potential therapeutic use of NIBS in clinical populations, particularly in those in which cortical excitability may have been altered chronically from normal levels. Specifically, applying what we have learned from studies of single session TMS/tDCS in healthy adults may not reflect what occurs in patient groups and/or following multiple sessions. Applying assumptions of frequency/polarity specific effects demonstrated in healthy adults could be problematic for clinical groups, particularly when previous research clearly indicates altered plasticity within TS. Suppa et al. (2011) found that in contrast to control subjects, TS participants showed no change in cortical excitability following single sessions of iTBS and cTBS. Wu and Gilbert (2012) also found TS participants showed reduced alterations in cortical excitability immediately following iTBS; and Brandt et al. (2014) reported reduced response to paired associative stimulation (PAS). Differences in the balance between physiological excitation and inhibition in TS (Jackson et al. 2015) and related conditions such as OCD and major depressive disorder (MDD) (Radhu et al. 2013) are likely, and an increasing amount of work suggests that altered homeostatic plasticity (which maintains the equilibrium of neural activity) may be a feature of several clinical conditions (Karabanov et al. 2015; Wondolowski and Dickman 2013). These factors are highly likely to impact on NIBS outcomes and the degree to which changes in synaptic plasticity can be achieved. With a few notable exceptions (Kahl et al. 2021; Wu et al. 2014), a few studies have sought to assess physiological changes occurring following NIBS interventions. Adopting a multi-faceted approach to therapeutic NIBS trials in which neuroimaging is incorporated into the design will facilitate the goal of establishing not only for who NIBS works for but also why. Specifically, thorough assessment of baseline states, connectivity, and neural anatomy may provide useful information in predicting positive outcomes.

In addition, although TMS and tDCS have been shown to be safe for pediatric populations (Allen et al. 2017; Palm et al. 2016), there remain considerations regarding the use of NIBS in this group. This may include the use of optimised stimulation protocols which differ from those used in healthy adults (Hameed et al. 2017). Extensive further research is likely needed to better understand synaptic plasticity and the effects of NIBS during typical and atypical development.

Understanding individual responses and being able to predict those who could benefit from NIBS is a critical step towards it becoming a useful therapy for TS and other conditions. The success of studies aiming to identify biomarkers associated with symptom reduction following NIBS will be dependent on having adequate statistical power, and thus, sample size will be a key factor. Understandably, therapeutic NIBS studies can be particularly difficult to recruit for, given the experimental nature of the treatment and the need for frequent visitation to treatment centres amongst other factors. A potential practical solution to this is to establish multi-site collaborations in which a standardized protocol is applied across numerous centres; alternatively, researchers could attempt and report internal replications. In the case of tDCS, studies making use of home application may be a feasible way of increasing sample size; however, it must be acknowledged that while home-use studies have increased ecological validity, a degree of experimental control is likely sacrificed.

### Optimising stimulation parameters

As noted above, therapeutic NIBS studies tend to select stimulation parameters based on results from research conducted in groups of healthy individuals, most often adults. These studies often involve applying the same stimulation to all individuals, an approach which ignores functional and anatomical differences, and is likely to minimise clinical outcomes. With rTMS/TBS approaches, some degree of personalization is possible with respect to the intensity and type of stimulation to be used. This is typically chosen based on a percentage of an individual's motor threshold (MT), measured as the minimal TMS intensity which can be used to induce a small muscle twitch within the hand when TMS is applied to the relevant location in motor cortex. MT is known to be heavily dependent on structural features including orientation of white matter fibres and skull-to-cortex distance (Cukic et al. 2009; Herbsman et al. 2009), and hence, using this approach, it is possible to address important anatomical inter-subject differences. However, given that both factors will differ depending on the cortical site, this approach is imperfect. Approaches to adjusting MT for cortical regions outside of PMC have been developed (for example Stokes et al. (2005) and should be considered when targeting non-motor regions. Localization of optimal stimulation sites is another aspect of rTMS/TBS work which could be improved in several studies. To ensure adequate coil localization, use of neuro-navigation systems is highly recommended. Preferably, this should be achieved by acquiring an anatomical scan for each participant and creating personalised targets (Julkunen et al. 2009), and when possible, the use of a task relevant fMRI localiser has also been shown to be beneficial in comparison to anatomical scan alone (Sack et al. 2009).

Unlike TMS approaches, tDCS does not induce action potentials and hence does not generate MEPs, even when applied over motor regions. This makes tailoring tDCS approaches more challenging, and as a result, many studies select a fixed intensity and a set electrode montage for all participants. One emerging approach for tailoring tDCS involves the use of computational current flow modelling (Bikson et al. 2012a, b; Sadleir et al. 2010). Several studies have suggested improved cortical targeting can be achieved through modelling current flow when considering optimal stimulation sites for the treatment of depression (Bai et al. 2014; Csifcsak et al. 2018) and stroke (Galletta et al. 2015). This is yet to be explored in TS research but could prove beneficial in optimising electrode placement.

### Choice of outcome measures

As is apparent in Tables 1 and 2, the outcome measures used to assess changes following NIBS in TS are diverse. Many of these are self-report questionnaires and semi-structured interviews which aim to capture changes in core tic symptoms and related co-morbidities. These outcome measures are critical given that the aim of such studies is to produce a meaningful reduction in clinical symptoms. However, while measuring change at a behavioural level is clearly the optimal outcome clinically, it does not aid our understanding regarding what neurobiological alterations may underpin these effects. Depending on the measures used, it is also often unclear if it is tic expression that is changed or the urge-to-tic that has been modulated, or both. Given that many measures are self-report, and many studies are not sham controlled, it is also important to consider the potential impact of unconscious biases and placebo effects in these measures. It is also important that measures are able to capture the variable nature of tics which are known to wax and wane over time (Peterson and Leckman 1998). The use of video recording in addition to questionnaire measures can be helpful in addressing this issue, as it gives a more objective view of changes in tic frequency and intensity. However, this is also limited as it only captures a snapshot of tics present in that moment, and these are likely to vary across situations. The YGTSS is also limited in that its original purpose is to gauge tics that have occurred during the last week. This may make it insensitive to changes occurring on a shorter time scale. Careful consideration of outcome measure and, when possible, the use of neuroimaging approaches will be critical in understanding which individuals might benefit from what NIBS approaches, and how these benefits manifest. Specifically, the addition of appropriate neuroimaging methods will allow researchers to quantify physiological responses to NIBS including the direction and magnitude of effects, which will allow further exploration into how these interplay with changes in symptoms.

## Conclusions

The evidence to date suggests that TMS and tDCS approaches may be helpful in reducing tics, yet there remains a substantial amount of further work needed for these approaches to reach a convincing level of supporting evidence. Complex interactions between clinical symptoms, cortical states, and the NIBS parameters require serious consideration, and the field urgently needs additional studies to address a number of issues.

Our recommendations for future work exploring the therapeutic impact of NIBS in TS are as follows:

1. There is a clear and urgent need for randomised sham-controlled studies with increased sample sizes. Ideally, sample sizes should be sufficiently large to allow for subgroup analysis. Exploration of interactions with medication use, co-morbidity, and symptom severity would be beneficial.
2. Researchers and clinicians should be mindful that the effects of a single stimulation session in healthy adults may not be the same as in clinical groups, in the developing brain, or following multiple sessions. Consideration should be given to the potential impact of cortical state prior to stimulation (e.g., during tic suppression) and the potential benefits of pairing NIBS with a therapeutically relevant behavioural task.
3. When possible, the adoption of a multimodal approach is desirable. This could help to reveal potential biomarkers of NIBS effectiveness and clinical outcomes.
4. Careful consideration should be given to the selection of TMS and tDCS parameters, and whenever possible, these should be tailored to individuals. This may involve the use of scaling metrics and/or the use of computational current flow modelling. In TMS studies, the use of neuro-navigation systems is recommended, ideally in conjunction with individual anatomical scans and functional localisers.
5. Outcome measures should be carefully considered and aim to capture a true reflection of alterations in tics, urges, and co-morbid symptoms/conditions over a sustained period.
